# Severe Fatal Mucormycosis in a Patient with Chronic Lymphocytic Leukaemia Treated with Zanubrutinib: A Case Report and Review of the Literature

**DOI:** 10.3390/curroncol30090599

**Published:** 2023-09-07

**Authors:** Giuseppe Maggioni, Marny Fedrigo, Andrea Visentin, Elisa Carturan, Valeria Ruocco, Livio Trentin, Mauro Alaibac, Annalisa Angelini

**Affiliations:** 1 Pathology Unit, Department of Medicine, University of Padova, Via A. Gabelli 61, 35121 Padova, Italy; 2 Cardiovascular Pathology Unit, Department of Cardio-Thoracic-Vascular Sciences and Public Health, University of Padova, 35128 Padova, Italy; 3 Hematology Unit, Department of Medicine, University of Padova, Via N. Giustiniani 2, 35128 Padova, Italy; 4 Dermatology Unit, Department of Medicine, University of Padova, 35128 Padova, Italy

**Keywords:** case report, mucormycosis, histopathology, haematological malignancy, CLL

## Abstract

Severe mucormycosis is a fatal disease rarely complicating chronic lymphoproliferative disorders. We present a fulminant and fatal case of a 74-year-old Caucasian woman suffering from CLL treated with second-generation BTK inhibitor zanubrutinib. After a first septic episode a month prior, originating from the lung with later systemic involvement by an unidentified agent and treated with large-spectrum antibiotics and fluconazonle, a slow-onset enlarging tender warm and erythematous nodular swollen cutaneous lesion appeared in her lower limbs and spread subsequently to her upper limbs, progressing towards central ulceration with a necrotic core. Suspecting a mycotic dissemination from an unknown agent, a skin punch biopsy was performed, and intraconazole was started. Due to spread of the skin lesions, the patient was hospitalized and intravenous liposomal ampthotericin B was started. Histopathology showed an atypical sporangium-rich mycotic angioinvasion of the small vessels. Only the increase of BDG and GM could corroborate the hypothesis of mycotic infection. However, long-term CLL, immunosuppressive therapies, neutropenia, and prior use of azoles and other antimycotic agents were risk factors for mucormycosis; BTK inhibitor could also be added as another novel risk factor. Despite all therapeutic efforts, the patient died. Post-mortem molecular exams confirmed the diagnosis of disseminated mucormycosis.

## 1. Introduction

Chronic lymphocytic leukaemia (CLL) is a haematological malignancy characterized by the clonal proliferation of long-lived CD5^+^ B lymphocytes in spleen and lymph nodes, bone marrow, and peripheral blood [1]. It is often associated with immune system dysregulation [2], which represents a contributing factor in the overall clinical picture.

The immune system dysregulation could be at least partly explained by CLL cells expressing high levels of the immune-suppressing cytokines, including TGF-β and IL-10 [3], low levels of adhesion and costimulatory molecules essential for the induction of effective immune responses, and an increased numbers of regulatory T-lymphocytes [4]. Other relevant elements are the alteration of both node and bone marrow microenvironments, creating a niche favouring the survival of neoplastic cells and impinging the immune responses caused by T-lymphocytes [5]. In addition, T-lymphocytes display markers of immune exhaustion, such as PD-1 (programmed cell death protein (1), that hinder their activation and function against the neoplastic cells [6]. Hypogammaglobulinemia is another common clinical characteristic of the CLL immune dysregulation. It can be found in almost 25% of the patients at diagnosis and up to 85% of relapsed and heavily treated cases [7]. It is usually a non-reversible phenomenon that increases the risk of infection. Infections are still the main cause of death among patients with CLL, accounting for 25–50% of the mortality rate in these patients [8,9]. The abovementioned immune dysregulation is further worsened by medical treatments, such as not chemoimmunotherapy, but also Bruton’s tyrosine kinase (BTK) inhibitors. This novel class of drugs revolutionized the treatment landscape for patients with CLL. BTK is a cytoplasmic tyrosine kinase that is important in B-lymphocyte development, survival, activation, and differentiation [10], and its dysregulation is a CLL pathogenic hallmark [1]. These drugs are meant to disrupt the aberrant activation of the B-cell receptor (BCR), leading to cell death [11]. However, these inhibitors can also modulate the immune microenvironment, shaping the activity of several immune cells, impacting an already-altered immune system, and raising concerns about their potential effects on susceptibility to infections, including mycetes. BTK inhibitors may impair innate immunity [12] and adaptive immunity and more specifically, BTK inhibition may impair T-lymphocyte function, shifting to a marked Th-1 selection with lower antibody production, and lower immune surveillance against pathogens [13]. Moreover, BTK also has a pivotal role in other immune cell populations, including macrophages, where it regulates receptor-mediated phagocytosis, including the phagocytosis of fungal organisms such as *Candida albicans* [14]. However, the infectious risks associated with BTK pharmacologic blockade remain poorly defined, despite reports of opportunistic infections in patients who received ibrutinib treatment, including cases of *Pneumocystis jirovecii* pneumonia (PJP), cryptococcosis, and invasive mould infection (including *Mucorales*), are becoming progressively more common [15]. BTK inhibitors could be therefore potentially considered a risk factor of infectious complications in CLL patients [12,16,17,18,19].

Given the previous points and the wide clinical use of BTK inhibitors, physicians should be aware of the risk to develop severe invasive mycosis.

Apart from the more common and well-documented infections by *Candida* and *Aspergillus* spp., mycetes belonging to the order of *Mucorales* represent a major clinical issue in CLL. Fungi belonging to this recently re-classified order [20,21,22,23] account for 38 species (out of 261 total) in 55 genera that have been reported to be responsible for clinically relevant pathologies in human patients [23,24]. The most prevalent genus causing clinical mucormycosis is *Rhizopus* (two main species) [25], followed by the genus *Mucor* (twelve species up to date) [24], *Lichtheimia*, and *Rhizomucor*; other less frequent pathogenic species are occasionally reported [26,27]. The fast growth, airborne spores, and the thermotolerance of these ubiquitarian saprotroph moulds enable them to grow at human body temperature: these features explain their pathogenic activity in patients with specific risk factors, including SARS-CoV-2 infection and treatments [28,29], as listed in Table 1 [20,26,30].

Little is known so far about these fungal cell structures, especially regarding the components of their cell wall [36] as well as their biology and metabolism, and this has a major clinically negative impact, both on the diagnostic side (laboratory tests on serum or other human fluids) and on the therapeutic side (the correct administration of specific drugs). Two main serological assays are used to detect mycosis: (1-3)-β-D-glucan (BDG) and galactomannan (GM). Both of these target key polysaccharide components of the fungal cell wall. These tests are particularly useful in a couple of specific situations: neutropenic fever due to hematologic malignancy and patients receiving transplants [37,38,39,40]. However, as stated in an important retrospective paper by Millon et al., GM and BDG tests display a low sensibility during mucormycosis, and tissue cultures take several days and are often negative, preventing early management [41]. The epidemiology of mucormycosis is complex to estimate, but it seems underrated even though there are studies showing growth, probably due to the increasing number of immunocompromised patients [42].

The clinical landscape of mucormycosis is heterogeneous. The infection is classified based on the anatomical site: rhino-orbito-cerebral (ROCM), pulmonary, gastrointestinal [43], cutaneous, and other miscellaneous [25,31,44]. They are also stratified according to the underlying risk factor [45].

Cutaneous mucormycosis can be seen not only in immunocompromised hosts, but also in immunocompetent patients [35,46,47]. The most common aetiology, regardless of the risk factors, is penetrating trauma, followed by iatrogenic lesions, including intramuscular injections and open wound trauma.

Based on the extent of invasion, cutaneous mucormycosis can be classified as a localized infection, deep extension, or a specific localization of a disseminated infection. A localized infection is seen in 32–56% of patients, usually restricted to the cutaneous and subcutaneous tissue without invading adjacent sites. Deep extension refers to the invasion of muscles, bones, and tendons, occurring in 24–52% of patients. In these cases, the infection often presents as necrotizing fasciitis with erythematous necrotic eschar. Cutaneous mucormycosis as part of a disseminated infection and refers to an infection involving other non-contiguous sites besides the cutaneous site, and is seen in 16–20% of cutaneous infections [44]. These infections are extremely burdensome for the healthcare system, leading to prolonged hospitalization and increased healthcare costs [48,49].

Here, we present the case of a fulminant fatal cutaneous mould infection in a 74-year-old Caucasian female patient suffering from chronic lymphocytic leukaemia (CLL).

## 2. Detailed Case Description

A 74-year-old female patient, a retired employee, was admitted to the haematology ward on 4 July 2020 after a progressive decline in her clinical condition. The patient lived alone and denied any foreign travel, skin trauma, abrasions, or the possession of pets. Apart from a less significant femoral head necrosis with an arthroprosthesis, her medical history was positive for CLL since 2006, characterized by a complex karyotype, TP53 mutation, and trisomy 12 at FISH [50,51]. She had undergone several lines of therapy, as listed in Table 2. She also had hypogammaglobulinemia, for which she was receiving subcutaneous immunoglobulins, but no diabetes mellitus.

Her medical history was complicated by several hospitalizations due to infections, including H1N1 influenza in 2016, primarily related to her immunosuppressive state and bronchiectasis.

Approximately one month prior (towards the end of May 2022), the patient was admitted to hospital due to pulmonary sepsis caused by an unidentified agent. The condition was managed with ceftriaxone, levofloxacin, and fluconazole. After an infectious disease consultation, the treatment was switched to meropenem, daptomycin, and caspofungin, leading to complete resolution.

**Figure 1:**
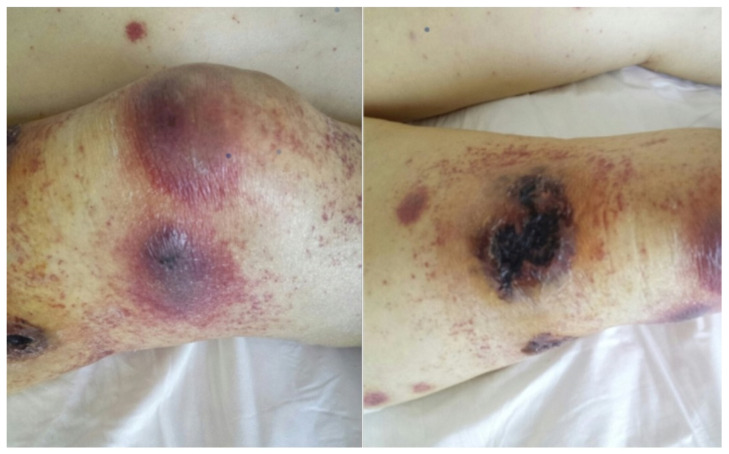
Skin lesions (**on the left**) showing the tender erythematous lesions and (**on the right**) necrotizing aspects.

Immediately after the discharge (4 June) blood tests showed a marked and gradual increase in C-reactive protein (CRP) levels along with concomitant grade 4 neutropenia (i.e., <500/μL) lasting for more than 15 days. The patient also experienced a subsequent febrile peak. Starting from mid-June (referring to the period from 15 June on), tender, warm, erythematous nodular swollen cutaneous lesion appeared in the lower limbs and subsequently also spread to the upper limbs. These lesions exhibited central ulceration with a necrotic core as they progressed (Figure 1).

In suspicion of a fungal infection, a lesion biopsy was performed on 25 June. Zanubrutinib was discontinued, and empirical therapy with oral itraconazole was initiated immediately after the biopsy.

A 5 mm Ø punch biopsy of the cutis and subcutis was conducted on the left thigh at the interface of the necrotic core and the macroscopically intact cutis. The biopsy specimen was fixed in a 10% neutral formalin solution. Macroscopic examination revealed dark skin pigmentation along with a 1 mm brownish nodular lesion without any other relevant features.

**Figure 2:**
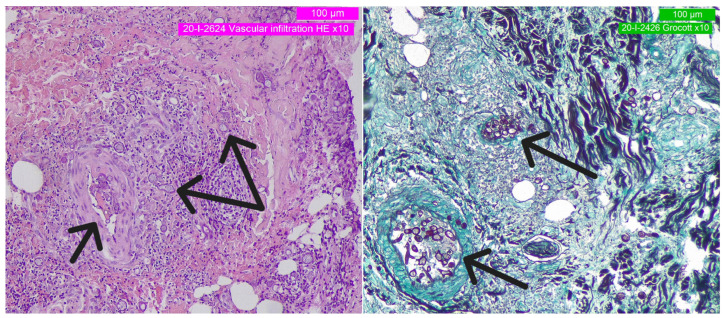
Slide panoramic showing the angioinvasive fungal mould. Black arrows indicate the hyphae **(on the left**: HE-10×; **on the right**: GMS-10×).

**Figure 3:**
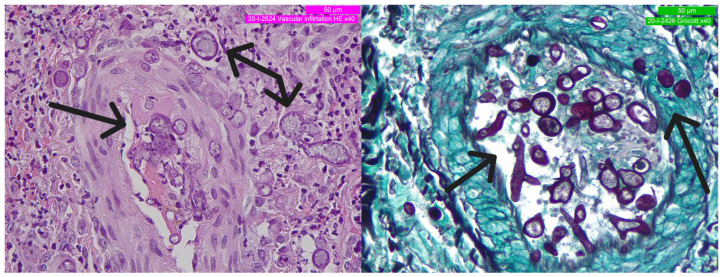
Details of fungal and vascular structures. Black arrows indicate the hyphae and sporangial bodies (**on the left**: HE-40×; **on the right**: GMS-40×).

In total, four stains were applied in addition to standard HE staining: Giemsa, GMS (Grocott–Gömöri methenamine silver stain), PAS (Periodic acid–Schiff), and Gram staining. Photographic documentation of the resulting slides was captured at various magnifications (see Figure 2 and Figure 3).

Microscopic analysis highlighted a severe, diffuse lympho-monocytic inflammation. Minimal to no necrotic material was observed. Sparse microbial structures exhibited a typical angioinvasive pattern, including thrombosed arterioles and small blood vessels. Hyphal mats and sporangial-type cells (specialized cells forming spores) were identified. Inside the vessels, a minute pauciseptate (coenocytic), thin-walled (5–15 µm), and minimally branching (at most) hyphal mat formed the primary histopathological finding. Additionally, numerous thick-walled ovoidal or spheroidal (5–20 µm) sporangia were detected within the relatively scarce vegetative hyphae. These thick-walled structures strongly stained with PAS, GMS, and Giemsa. Slide examination immediately disclosed many critical points, due to the aggressive presentation of a rare cutaneous infection from an unknown site.

The case was extensively discussed, and specialized Veterinary Pathologists from our University were consulted. Given the complexity of the case and the lack of positive laboratory evidence, only a presumptive diagnosis of deep cutaneous fungal infection was reached. The anatomopathological report listed rhinococcidioidomycosis, chromoblastomycosis, or scytalidosis as potential diagnostic alternatives.

In suspect of severe mould infection, clinical findings and X-ray study of the chest were conducted, but they excluded local recurrences of infection. Furthermore, sinonasal clinical findings were negative. Itraconazole treatment proved ineffective, and cutaneous nodules increased after 10 days of therapy. The patient was hospitalized and was managed with liposomal amphotericin B (L-AmB) 10 mg/kg daily. Although this approach slowed down the progression of cutaneous lesions, a consistent increase in BDG and GM was observed from the first day, suggesting a potential co-infective progression to, likely, mycotic bronchopneumonia. In such a disseminated form of disease, the mortality rate is exceedingly high [52].

Unfortunately, both pathogen culture from the cutaneous scratch and multiple-site repeated blood cultures yielded no positive results. While Mucorales species are known as angioinvasive fungi, positive blood culture results are infrequent unless there is luminal involvement of a vascular catheter [53].

Ultimately, despite all therapeutic interventions attempted, the patient passed away after 30 days of hospitalization. No autopsy was required by physicians.

## 3. Discussion

Invasive fungal infections are rare in patients with lymphoproliferative disease like CLL [54,55], although they are associated with a dismal prognosis [56]. While BTK inhibitors have improved the treatment landscape for CLL patients, they have been linked to adverse events, such as atrial fibrillation, bleeding, diarrhoea, and infections [57,58]. Despite their selectivity towards BTK, these inhibitors can also impact other kinases, such as ITK, EGFR, and TEC, leading to adverse effects. BTK inhibitors like ibrutinib and zanubrutinib have been reported to impair the innate response against fungal infections by affecting BTK in macrophages [59].

Following the patient’s demise, the case was completely re-examined histologically and subsequently discussed collectively, taking into account the entire clinical history. Molecular analysis through DNA extraction became the final step in establishing the definitive, accurate diagnosis of an invasive fungal infection (IFI) caused by invasive mucormycosis.

There are a few issues related to this case which should be addressed.

The clinical diagnosis of fungal infection in this case was challenging. First of all, the distinctive type of infection prompted the consideration of parasitic diseases, including rare ones. The exclusion of both unicellular and multicellular parasites was guided by two main indicators: the pronounced positivity of cell walls with GMS staining and serological positivity for fungal markers.

Despite its relative weakness, GM positivity in laboratory tests (a week after the biopsy) brought invasive aspergillosis into the differential diagnosis, given also the recent pneumonia. The mould appearance of aspergillosis is often mistaken for mucormycosis. The size of hyphae is usually an element of distinction, whereas hydropic and swollen aspergillar hyphae cannot be clearly distinguished from Mucorales hyphae. No primary focus of infection was identified; this complicated the identification of the pathogen, giving rise to doubts in the differential diagnosis. The histological elements were non-conclusive as well. Severe angioinvasive pattern is a pivotal element in disseminated mycosis. In disseminated aspergillosis, it is not uncommon to see hyphae invading dermal blood vessels walls, producing septic emboli and causing thrombosis and associated necrosis [60]. In retrospect, the branching at right angles and rare septation, as partially seen in our slides (Figure 3), could have possibly been considered a hint. Another critical histological element that misled the diagnosis towards coccidiodomycosis was the presence of numerous large sporangia. Nevertheless, this element was in contrast with the medical history of the patient, i.e., denying visiting foreign countries.

Serological fungal markers themselves provide little help in mucormycosis diagnosis [41], similarly to blood and tissue cultures. In fact, despite Mucorales species being highly angioinvasive fungi, even with the demonstration of positive histopathologic hyphae, blood cultures that allow for proper antibiograms only turn positive in about 50% of cases [53,61], as was in our case. New and promising techniques have been under development, including an ELISA test reacting for highly purified fucomannan wall carbohydrates of *Mucor* spp. [61,62].

Isolating the pathogen in a case with an uncertain diagnosis is essential for several reasons. This includes identifying potential sources of contamination within sensitive wards that house vulnerable patients and enhancing overall patient management. Moreover, recent studies highlight unpredictable antifungal susceptibility within the same Mucorales order [24], leading to unforeseeable drug combinations, like L-AmB and caspofungin. (This last one usually seen as ineffective based on clinical and laboratory experience due to its high minimal inhibiting concentration (MIC) [63,64]). Surprisingly, this combination has shown effectiveness not only in murine models [65], but also in promising case reports [66,67], even from highly endemic areas [28], despite being limited to ROCM and diabetic ketoacidosis patients [68].

Could a positive culture with a clear antibiogram have led to a more specific susceptibility-based therapy, potentially yielding a more favourable patient outcome, possibly through a combination of L-AmB with a more pathogen-targeted drug-like caspofungin?

The answer is not straightforward, as caspofungin’s role in this case is already contentious, considering it could be considered a potential risk factor for mucormycosis. The concurrent administration of chemotherapy and non-Mucorales-specific antifungals could facilitate species selection [35], as could have potentially happened during her last hospitalization.

These results are in contrast with studies examining antifungal combinations in HSCT and patients with haematological malignancies, which show no difference in mortality rates between combination and single-drug therapy [69]. The last guidelines (2019) underline the lack of definitive data in support of a combination therapy beyond a marginal recommendation. In this limited-data context, combinations of polyenes and azoles or polyenes plus echinocandins are mentioned [70], especially when referring to patients administered with targeted therapies (like our patient) [71]; however, the role of prophylactic azoles in these patients is still matter of dispute [72].

Lastly, the absence of an autopsy conducted by a pathologist was a limitation. Autopsies must be considered, especially in cases without a definitive diagnosis, providing pathologists with a specific clinical question to answer and the opportunity to obtain more suitable or abundant material for analysis.

## 4. Conclusions

Managing complex fungal infections in CLL patients can prove exceptionally challenging due to their rarity, the limited sensitivity of serological-microbiological techniques, and the limited specificity of histopathological examination of suspicious lesions. The thoughtful application of molecular biology techniques is crucial in achieving a precise diagnosis. Administering poly-antifungal therapies should always be approached with caution, as it can be difficult to accurately assess the risk-benefit ratio of these drugs in relation to the specific mycetes species involved.

## Figures and Tables

**Table 1:** Risk factors associated with mucormycosis.

Non-Immunological Risk Factors	Immunological Risk Factors	Special and Novel Risk Factors
Decompensated diabetes mellitus and ketoacidosis [31,32,33]	Immunodepression Primitive: solid and/or hematologic malignancies; autoimmunity	Premature neonates [34]
		Iron overload
		Major trauma
		Prolonged use of corticosteroids
		Intravenous drug abuse [27]
	Iatrogenic/secondary: hematopoietic stem cell (HSCT); solid organ transplant	Preventive or therapeutic antimycotic drugs (voriconazole, itraconazole, or caspofungin) [35]
		BTK inhibitor [12,15,16,17,18,19]
		SARS-CoV-2 infecion and treatment [28,29]

**Table 2:** Synopsis of antitumoral schedule.

2006	FCR protocol (Fludarabine-Cyclophosphamide-Rituximab)
2010	FCR
2012	Rituximab
2013	Bendamustine
2014–2016	Ibrutinib (discontinued due to infections)
2017–2020	Venetoclax
2020	Idelalisib-Rituximab
May 2020	Zanubrutinib

## Data Availability

No new data were created or analysed in this study. Data sharing is not applicable to this article.
